# Manifestation of malaria in Mangaluru, southern India

**DOI:** 10.1186/s12936-018-2462-7

**Published:** 2018-08-29

**Authors:** Prabhanjan P. Gai, Frank P. Mockenhaupt, Konrad Siegert, Jakob Wedam, Archith Boloor, Suyamindra S. Kulkarni, Rashmi Rasalkar, Arun Kumar, Animesh Jain, Chakrapani Mahabala, Pramod Gai, Shantaram Baliga, Rajeshwari Devi, Damodara Shenoy

**Affiliations:** 1 Institute of Tropical Medicine & International Health, Charité-University Medicine Berlin, Berlin, Germany; 2 Kasturba Medical College, Mangaluru, Manipal Academy of Higher Education, Manipal, Karnataka India; 3 Karnataka Institute for DNA Research, Dharwad, Karnataka India; 4 Wenlock Hospital, Mangaluru, Karnataka India; 5 District Vector Borne Disease Control Programme Office, Dakshina Kannada, Mangaluru, Karnataka India

**Keywords:** Malaria, India, Mangaluru, *Plasmodium vivax*, *Plasmodium falciparum*, Admission, Severe, Fatal

## Abstract

**Background:**

Severe and fatal vivax malaria is increasingly reported from India. In Mangaluru, southern India, malaria is focused in urban areas and associated with importation by migrant workers. In Wenlock Hospital, the largest governmental hospital, the clinical, parasitological and biochemical characteristics of malaria patients were assessed.

**Methods:**

During the peak malaria season in 2015 (June to December), outpatients were interviewed and clinically assessed. Malaria was ascertained by microscopy and PCR assays, concentrations of haemoglobin, creatinine and bilirubin, as well as thrombocyte count, were determined, and severe malaria was defined according to WHO criteria.

**Results:**

Among 909 malaria patients, the vast majority was male (93%), adult (median, 26 years) and of low socio-economic status. Roughly half of them were migrants from beyond the local Karnataka state, mostly from northern and northeastern states. Vivax malaria (69.6%) predominated over mixed *Plasmodium vivax*–*Plasmodium falciparum* infection (21.3%) and falciparum malaria (9.0%). The geometric mean parasite density was 3412/µL. As compared to vivax malaria, patients with falciparum malaria had higher parasite density and more frequently showed impaired general condition, affected consciousness and splenomegaly. Also, they tended to more commonly have anaemia and increased creatinine levels, and to be hospitalized (7.3%). Mixed-species infections largely assumed an interim position. Severe malaria (3.5%) was not associated with parasite species. No fatality occurred.

**Conclusion:**

In this study, uncomplicated cases of malaria predominated, with *P. falciparum* causing slightly more intense manifestation. Severe malaria was infrequent and fatalities absent. This contrasts with the reported pattern of manifestation in other parts of India, which requires the analysis of underlying causes.

## Background

India has achieved major reductions in the burden of malaria in the last decade. However, the country still contributes 6% of global malaria cases and accounts for approximately half of the total *Plasmodium vivax* cases worldwide [1, 2]. *Plasmodium vivax* has long been regarded a rather benign disease, irrespective of its substantial morbidity in Asia and Central and South America. However, severe vivax malaria has been increasingly reported in recent years, particularly from India [3–6]: among those hospitalized with *P. vivax* mono-infection, 11–45% developed severe malaria, including cerebral malaria and fatalities, in various settings in the country [7–9]. A recent systematic review and meta-analysis on severe vivax malaria since 1900 revealed that the majority of reports originated from India, that severe thrombocytopaenia (< 50,000/µL) was the most common defining symptom and that the overall case fatality rate was 0.3% [6]. Nevertheless, prevalence syndromes and fatality of severe vivax malaria appears to differ with defining criteria, age, endemicity and geographical setting, and rate of co-morbidities, among others [3, 6]. For instance, in one study from Western New Guinea, severe anaemia (haemoglobin (Hb) < 6 g/dL) was the predominant feature of severe vivax malaria [10], while respiratory distress was reported to predominate in neighbouring Papua New Guinea [11]. The role of mixed *P. vivax*–*P. falciparum* infections in terms of clinical manifestation and severity is not well established, not simply because of the very low sensitivity of conventional microscopy in detecting the minority species [12–14]. In mixed-species infections, both increased severity compared to *P. vivax* mono-infection but beneficial effects such as curbed peak *P. falciparum* parasite density have been observed [9, 11, 12, 15, 16].

In Mangaluru (population approximately 500,000), a harbour city in Karnataka, southern India, malaria shows particular characteristics. Located on the shores of the Arabian Sea with its hot and humid climate, Mangaluru receives an annual average of 3450 mm rainfall with one monsoon season between May and October [17]. Malaria episodes in the district reduced by two-thirds between 2005 and 2013 [18]. However, governmental records indicate a minimum of 6000 episodes of malaria per year in Mangaluru, with most cases occurring in urban rather than in rural areas. Among these, *P. vivax* dominates (82%), and migrant workers cause substantial importation of malaria from other parts of India [19, 20]. The urban nature of malaria in Mangaluru is largely attributed to the abundance of inner-city construction sites with stagnant water bodies, the migration of workers from malaria-endemic parts of India to work on these sites, and their poor housing conditions [19, 20]. *Anopheles stephensi* is the perdominant vector in this area [21].

Published data on the current clinical presentation of malaria in Mangaluru are virtually absent. The present study aimed at providing a description of the manifestation of malaria at the largest governmental health facility in Mangaluru, the 900-bed Wenlock Hospital, and to specifically assess differences between *P. vivax* and *P. falciparum* mono-infections as well as mixed-species infections.

## Methods

### Study site

Mangaluru (Mangalore) is a harbour city of 485,000 inhabitants (agglomeration, 624,000; 2011 national census data) located at the Arabian Sea in Karnataka, South India. Wenlock Hospital (900 beds) is the largest governmental hospital in Mangaluru offering treatment particularly for the economically deprived part of the population. In addition, several private hospitals provide health services in the absence of primary health care facilities in this urban setting. In 2014, Wenlock Hospital reported 6767 malaria cases, 80.1% being *P. vivax* mono-infections. Patients attending the outpatient department showing symptoms suspicious of malaria are directed to the hospital’s malaria diagnostic unit. From June to December 2015, during the peak malaria season, malaria patients were recruited at the malaria diagnostic unit during the operating hours of the outpatient department (08:00–16:00). Patients attending at other times were not considered. Patients confirmed to have malaria were treated according to standard guidelines on an outpatient basis, i.e., chloroquine for 3 days plus primaquine for 14 days for vivax malaria; artesunate–sulfadoxine–pyrimethamine for 3 days plus single dose primaquine on the second day in case of falciparum malaria; and, artesunate–sulfadoxine–pyrimethamine for 3 days plus primaquine for 14 days in case of mixed *P. vivax*–*P. falciparum* infection. Admission to ward was based on the attending physician’s discretion. Patients were enrolled into the study upon microscopic diagnosis of malaria, and all study participants provided informed written consent. The study protocol was reviewed and approved by the Institutional Ethics Committee of Kasturba Medical College, Mangaluru, Manipal University, and permission to conduct the study was granted by the Directorate of Health and Family Welfare Services, Government of Karnataka.

### Examinations

Upon recruitment, patients were interviewed using preformed questionnaires on socio-economic parameters, education, occupation, household characteristics, and malaria-related behaviour. A medical history was taken and a clinical examination was performed in all patients applying standardized documentation forms. Weight and height were measured. Body mass index (BMI) was calculated as kg/m^2^ and fever defined as axillary temperature ≥ 37.5 °C. Venous blood was collected into EDTA. Malaria parasites were counted per 200 white blood cells (WBCs) on Giemsa-stained thick blood films, and parasite species defined based on thin-film microscopy. Following DNA extraction (Qiamp blood mini kit, Qiagen, Germany), *Plasmodium* species was ascertained by nested polymerase chain reaction (PCR) assays [14]. Plasma was separated from blood by centrifugation. Routine hospital laboratory services provided results for haemoglobin (Hb) concentration (photometrically), WBCs and thrombocytes counts (Coulter principle) as well as concentrations of creatinine (Jaffé reaction), total bilirubin (DPD method), and direct bilirubin (Jendrassik–Grof method). Abnormal laboratory values were defined as: anaemia Hb < 11 g/dL (< 5 years), < 11.5 g/dL (5 to < 12 years), < 12 g/dL (12 to < 15 years, or females ≥ 15 years) and < 13 g/dL (males ≥ 15 years) [22]; leukocytosis > 10,000 WBCs/µL; thrombocytopaenia < 150,000/µL; increased creatinine > 1.4 mg/dL; increased total bilirubin > 1.2 mg/dL, and increased direct bilirubin > 0.2 mg/dL. Severe malaria was defined based on the current WHO definition [23] with some modifications. In particular, hypotension (systolic blood pressure < 80 mmHg in adults and < 70 mmHg in children) was considered a sign of severe malaria irrespective of the absent assessment of capillary refill or impaired perfusion; acidosis and hypoglycaemia were not routinely assessed; confusion in adult patients with a Glasgow coma score > 11 was also considered indicative of severe malaria. Of note, definitions of renal impairment (plasma creatinine > 3 mg/dL, or urea > 20 mM) and jaundice (plasma bilirubin > 3 mg/dL plus parasitaemia > 100,000/µL) followed WHO criteria as did the definition of severe malarial anaemia (Hb < 5 g/dL in children, or Hb < 7 g/dL in adults plus parasitaemia > 10,000/µL; no parasite density threshold for vivax malaria).

### Statistical analysis

Patients were considered for analysis if they had microscopically visible and PCR confirmed parasitaemia and, for admitted patients, if malaria diagnosis was available within 24 h. Data analysis was performed using SPSS 22 (IBM Corp., Armonk, NY, USA) and Statview 5.0 (SAS Institute Inc., Cary, NC, USA). Continuous parameters were compared between groups by Student’s *t* test, analysis of variance (ANOVA), Mann–Whitney U-test, or Kruskal–Wallis test as applicable. Proportions were compared between groups by Chi square (χ^2^) test or Fisher’s exact test, and odds ratios (ORs) and 95% confidence intervals (CIs) were calculated. Logistic regression was used to calculate adjusted odds ratios (aORs). Independent predictors of severe malaria were calculated by logistic regression analysis including factors showing association with severe malaria at a level of *P *< 0.10 and with backward removal of factors not associated in multivariate analysis (*P *> 0.05). A *P*-value < 0.05 was considered statistically significant.

## Results

A total of 909 patients with microscopically visible parasitaemia and PCR confirmed *Plasmodium* species were analysed (Table 1). Their median age was 26 years, the vast majority was male (92.8%), and most individuals (77.8%) had migrated to Mangaluru a median period of 6 months before presentation (range 1–600 days), predominately for working (96.0%; 680/708). Roughly half of the patients were migrants originating from beyond the state of Karnataka, and among them, most (82.8%, 371/448) were from northern and northeastern Indian states (West Bengal, Jharkhand, Uttar Pradesh, Bihar, Odisha, Assam). More than half of the patients (56.1%, 510/909) were either construction workers or daily labourers, and this figure was 76.3% (283/371) among the north/northeastern migrants. Most of the patients were from a low socio-economic status (SES) background (Table 1). More than two-thirds had incomplete or no formal education. The median monthly family income was approximately €80, on which a median of four individuals (1–15) were dependent. Although electricity was available in most households, household assets were limited. Approximately 40% of the patients stated using either bed net or repellent coils for malaria prevention. Almost half of the patients (45.8%, 415/906) reported to have had malaria before, the vast majority of those within the preceding year (82.7%, 343/415). Anti-malarial treatment (chloroquine and/or primaquine) within the preceding 4 weeks was reported by 0.6% (5/908) of patients and current medication by 1.3% (12/908; most commonly antihypertensives, 4; histamine-2 blockers, 4; antidiabetics, 3).

**Table 1:** Characteristics of 909 malaria patients from Mangaluru, India

Parameter	All	*P. vivax*	*P. vivax/P. falciparum*	*P. falciparum*	*P*
No. (%)	909 (100)	633 (69.6)	194 (21.3)	82 (9.0)	–
Age (years; median, range)	26.0 (4.0–82)	25.0 (4–70)	27.5 (7–82)	30.5 (12–65)	0.11
Male (%, n)	92.8 (844)	93.4 (591)	92.8 (180)	89.0 (73)	0.36
Migrated to Mangaluru (%, n)	77.8 (706/907)	77.3 (488/631)	80.4 (156)	75.6 (62)	0.58
Origin (%, n)					
Mangaluru	22.1 (201)	22.6 (143)	19.6 (38)	24.4 (20)	
Karnataka	27.3 (248)	25.3 (160)	31.4 (61)	32.9 (27)	
Non-Karnataka states	49.3 (448)	50.9 (322)	46.9 (91)	42.7 (35)	
No data/unclear	1.3 (12)	1.3 (8)	2.1 (4)	0	0.31
Self-defined tribal origin (%, n)	18.3 (166)	19.0 (120)	18.0 (35)	13.4 (11)	0.47
Formal education					
None	32.8 (298)	30.2 (191)	38.1 (74)*	40.2 (33)	
Incomplete (< 10th class)	38.0 (345)	39.2 (248)	37.1 (72)	30.5 (25)	
Completed (10th class)	13.9 (126)	15.0 (95)	10.8 (21)	12.2 (10)	
Advanced [(pre-)university]	14.6 (133)	15.3 (97)	12.4 (24)	14.6 (12)	
No data/unclear	0.8 (7)	0.3 (2)	1.5 (3)	2.4 (2)	0.06
Occupation construction worker or daily labourer (%, n)	56.1 (510)	53.7 (340)	58.8 (114)	68.3 (56)*	*0.03*
Monthly family income (rupees; median, range), n = 893	6000 (0–35,000)	7000 (0–35,000)	6000 (0–30,000)	6000 (2000–20,000)*^†^	*0.006*
Monthly family income < median (6000 rupees)	35.2 (314/893)	33.0 (205/621)	36.3 (69/190)	48.8 (40)*	*0.02*
People living in household (number; median, range), n = 889	5 (1–70)	5 (1–70)	5 (1–40)	4 (1–25)*^†^	*0.02*
People per room in household (number; median, range), n = 870	4 (0.25–70)	4 (0.25–70)	4 (0.50–40)	3 (0.67–25)*^†^	0.09
Household characteristics (%, n)					
Electricity	94.4 (857/908)	95.3 (602/632)	92.8 (180)	91.5 (75)	0.21
Electric fan	70.0 (636/908)	70.6 (446/632)	71.6 (139)	62.2 (51)	0.26
TV set	18.9 (172/908)	21.4 (135/632)	14.9 (29)	9.8 (8)*	*0.01*
Fridge	5.2 (47/908)	5.9 (37/632)	4.6 (9)	1.2 (1)	0.19
Motorbike	3.6 (33/908)	4.9 (31/632)	1.0 (2)*	0*	*0.008*
Radio	2.8 (25/908)	3.0 (19/632)	1.5 (3)	3.7 (3)	0.48
Bicycle	1.7 (15/908)	1.7 (11/632)	1.5 (3)	1.2 (1)	0.93
Stated use of a bed net in preceding night (%, n)	39.1 (354/906)	41.0 (259/631)	35.2 (68/193)	32.9 (27)	0.17
Window nets present (%, n)	4.2 (38/906)	4.0 (25/631)	6.7 (13/193)	0^†^	*0.04*
IRS in preceding 6 months (%, n)	2.8 (25/906)	3.3 (21/631)	1.6 (3/193)	1.2 (1)	0.28
Stated use of repellent coils (%, n)	39.3 (356/906)	39.6 (250/631)	40.4 (78/193)	34.1 (28)	0.59
Stated use of repellent fluids (%, n)	9.7 (88/906)	10.0 (63/631)	8.3 (16/193)	11.0 (9)	0.72
Stated stagnant water bodies at home (%, n)	31.0 (281/906)	32.0 (202/631)	27.5 (53/193)	31.7 (26)	0.48
Stated malaria episode in preceding 12 months (%, n)	37.9 (343/906)	38.8 (245/631)	34.2 (66/193)	39.0 (32)	0.50
Time since last malaria episode (months; median, range), *n *= 415	5 (0.2–240)	4 (0.2–180)	6 (0.5–240)	9 (0.5–120)	0.19

* P < 0.05 as compared to vivax malaria; ^†^ P < 0.05 as compared to mixed species malaria

Most of the patients had vivax malaria (69.6%, 633/909), 21.3% (194/909) harboured both *P. vivax* and *P. falciparum*, and 9.0% (82/909) of individuals had falciparum malaria. However, these proportions changed over time.

**Fig. 1:**
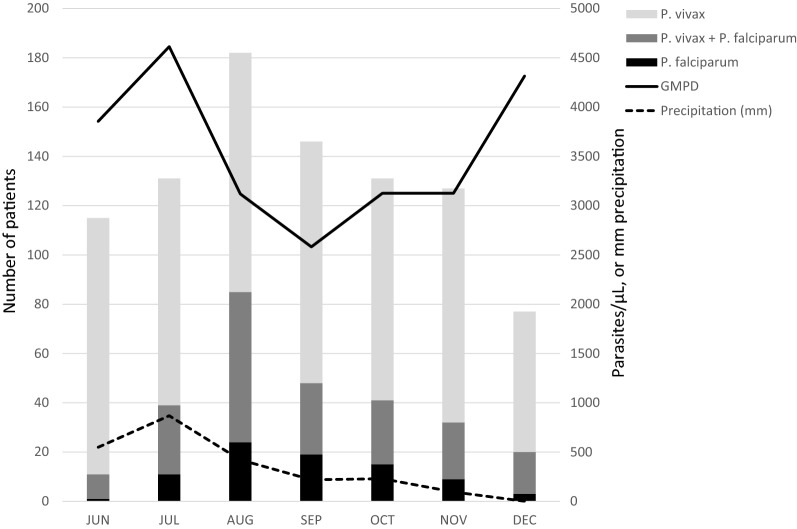
Parasite species, geometric mean parasite density and precipitation according to month of presentation

Precipitation was highest in June to August 2015 (1836 mm) and declined thereafter in September to December (543 mm). In parallel, the proportion of *P. vivax* mono-infection declined from 90.4% (104/115) in June to a low of 53.3% (97/182) in August and increased thereafter (Fig. 1). Socio-demographic factors had limited influence on parasite species. As compared to patients with *P. vivax* or mixed species infections, those with falciparum malaria tended to be older and to less commonly have formal education. The proportion of low income was highest among falciparum malaria patients and ownership of wealth indicators (e.g., TV set, motorbike) was lowest. Migration and origin did not overtly affect parasite species; nevertheless, *P. falciparum* mono-infection tended to be less common in patients originating from outside the state of Karnataka (OR, 0.75 (95% CI, 0.46–1.21), *P *= 0.21, Table 1) and this difference was statistically significant for north/northeastern migrants (5.4% (20/371) vs 11.5% (62/538); OR, 0.44 (95% CI, 0.25–0.76), *P *= 0.002). At the same time, falciparum malaria was comparatively common among construction workers/daily labourers (Table 1). In multivariate analysis, including origin and occupation (and month of recruitment), falciparum malaria was positively associated with being a construction workers/daily labourer (aOR, 2.49 (95% CI, 1.49–4.17), *P *= 0.0005) and negatively with migration from North/northeastern India (aOR, 0.33 (95% CI, 0.19–0.58), *P *= 0.0001). Malaria prevention, with the potential exception of window nets, as well as previous (Table 1) or recent (Table 2) malaria episodes did not influence parasite species.

**Table 2:** Patients’ history according to malaria parasite species

Parameter	*P. vivax*	*P. vivax/P. falciparum*	*P. falciparum*	*P*
No. (%)	633 (69.6)	194 (21.3)	82 (9.0)	–
Stated history of malaria within preceding 1.5 months (%, n)	6.2 (39)	5.7 (11)	9.8 (8)	0.41
Stated duration of current episode (days; median, range)	3.0 (1–30)	3.0 (1–15)	3.0 (1–20)	0.55
Stated current signs and symptoms (%, n)				
Fever	99.7 (630/632)	99.5 (193)	100 (82)	0.79
Headache	93.5 (591/632)	95.4 (185)	96.3 (79)	0.43
Chills/shivering	88.4 (559/632)	90.7 (176)	82.9 (68)	0.18
Fatigue/weakness	86.1 (544/632)	89.7 (174)	90.2 (74)	0.29
Muscle pain	85.6 (541/632)	86.1 (167)	89.0 (73)	0.70
Sweats	75.6 (477/632)	71.5 (138)	69.5 (57)	0.31
Back pain	69.6 (440/632)	74.7 (145)	72.0 (59)	0.38
Cough	42.9 (271/632)	41.8 (81)	43.9 (36)	0.94
Nausea	41.0 (259/632)	44.0 (85/193)	41.5 (34)	0.75
Abdominal pain	30.4 (192/632)	32.0 (62)	37.8 (31)	0.39
Vomiting	27.4 (173/632)	34.5 (67)	36.6 (30)	0.06
Dyspnoea	7.1 (45/632)	3.1 (6)*	6.1 (5)	0.13
Diarrhoea	4.0 (25/632)	3.1 (6)	4.9 (4)	0.76
Stated co-morbidities (%, n)				
Diabetes mellitus	1.7 (11/632)	0.5 (1)	1.2 (1)	0.45
HIV/AIDS	0.3 (2/632)	0	0	0.65
COPD	0.6 (4/632)	0	0	0.42
Hypertension	0.8 (5/632)	3.1 (6)*	0	*0.02*
Clinical malnutrition	1.1 (7/632)	0.5 (1)	0	0.50
Chronic liver disease	0.3 (2/632)	0.5 (1)	0	0.79

* P < 0.05 as compared to vivax malaria; ^†^ P < 0.05 as compared to mixed species malaria

The geometric mean parasite density (GMPD) was 3412/µL (95% CI, 3081–3779), and it was significantly higher in *P. falciparum* and mixed-species infections than in vivax malaria (Table 3). The median duration of disease preceding presentation was 3 days (range 1–30), without differences according to parasite species (Table 2). Reported signs and symptoms did not differ with respect to parasite species even though sweating tended to be more commonly reported by vivax malaria patients as compared to patients with mixed species or falciparum malaria, and vomiting and abdominal pain was less commonly reported (Table 2). Only a few patients reported existing co-morbidities.

**Table 3:** Clinical and laboratory characteristics according to parasite species

Parameter	*P. vivax*	*P. vivax/P. falciparum*	*P. falciparum*	*P*
No. (%)	633 (69.6)	194 (21.3)	82 (9.0)	–
GMPD (/µL; 95% CI)	2999 (2660–3382)	4246 (3413–5283)*	5408 (3758–7780)*	*0.0005*
General condition, impaired (%, n)	4.3 (27/628)	4.7 (9/192)	16.0 (13/81)*^†^	*<* *0.0001*
Consciousness affected (%, n)	2.2 (14/629)	3.6 (7/192)	11.1 (9/81)*^†^	*0.0001*
Splenomegaly (%, n)	16.6 (104/628)	20.1 (39)	29.6 (24/81)*	*0.01*
Petechia (%, n)	1.0 (6/629)	0.5 (1)	2.4 (1)	0.33
Admission to ward (%, n)	3.3 (21)	4.1 (8)	7.3 (6)	0.20
Axillary temperature (°C, mean ± SD), n = 903	37.1 ± 1.5	37.4 ± 1.5*	37.3 ± 1.7	*0.03*
Weight (kg; median, range), n = 899	54.5 (11.4–96.8)	52.8 (17.2–89.9)	52.0 (31.6–71.7)*	*0.03*
Height (cm, mean ± SD), n = 889	165.0 ± 10.4	165.0 ± 10.0	165.5 ± 8.6	0.92
BMI (kg/m^2^; median, range), *n* = 887	19.8 (12.3–39.5)	19.1 (12.2–46.4)	19.2 (12.7–27.6)	*0.002*
Heart rate (/min; median, range), n = 906	99.0 (45–198)	100.0 (56–145)	104.5 (59–140)	0.16
Blood pressure, systolic (mmHg; median, range), n = 908	116.0 (71–250)	116.0 (66–217)	115.5 (78–194)	0.41
Blood pressure, diastolic (mmHg; median, range), n = 908	74.0 (27–155)	75.0 (40–161)	73 (42–109)	0.54
Hypotension (systolic BP < 80 mmHg; %, n)	1.1 (7/632)	3.6 (7)*	1.2 (1)	*0.05*
Respiratory rate (/min; median, range), n = 900	24.0 (14–44)	24.0 (15–34)	24.0 (16–44)	0.19
Thrombocytes (/µL; median, range), n = 859	110,000 (4000–326,000)	96,000 (10,600–293,000)	106,000 (6000–658,000)	0.28
Thrombocytopaenia (< 150,000/µL; %, n)	72.5 (437/603)	77.0 (141/183)	74.0 (54/73)	0.47
Severe thrombocytopaenia (< 50,000/µL; %, n)	10.9 (66/603)	14.2 (26/183)	19.2 (14/73)*	0.09
Hb (g/dL; median, range), n = 869	13.7 (5.1–20.3)	13.1 (7.0–20.5)*	13.5 (5.0–18.4)	*0.02*
Anaemia (%, n)	30.8 (188/610)	44.1 (82/186)*	41.1 (30/73)	*0.002*
Severe malarial anaemia (%, n)	0.7 (4/610)	0	0	0.43
White blood cells (/µL; median, range), n = 849	5400 (600–20,700)	5350 (700–17,400)	5500 (2900–16,400)	0.43
Creatinine (mg/dL; median, range), n = 828	0.90 (0.30–6.70)	0.90 (0.40–2.40)	0.90 (0.50–1.90)	0.73
Increased creatinine (> 1.4 mg/dL; %, n)	2.4 (14/576)	3.9 (7/179)	5.5 (4/73)	0.26
Renal impairment (creatinine > 3 mg/dL)	0.9 (5/576)	0	0	0.33
Bilirubin, total (mg/dL; median, range), n = 832	1.19 (0.20–8.10)	1.16 (0.30–8.72)	1.33 (0.37–5.85)	0.54
Increased bilirubin (> 1.2 mg/dL; %, n)	48.3 (280/580)	46.7 (84/180)	52.8 (38/72)	0.68
Bilirubin, direct (mg/dL; median, range), n = 833	0.21 (0.01–6.05)	0.22 (0.09–4.01)	0.25 (0.09–4.86)	0.41
Increased direct bilirubin (> 0.2 mg/dL; %, n)	50.0 (290/580)	51.4 (93/181)	55.6 (40/72)	0.66
Jaundice (bilirubin > 3 mg/dL and > 100,000 parasites/µL)	0	0.6 (1/181)	0	0.16

* P < 0.05 as compared to vivax malaria; ^†^ P < 0.05 as compared to mixed species malaria

Upon clinical examination, patients with falciparum malaria showed increased proportions of impaired general condition, affected consciousness and splenomegaly (Table 3). Accordingly, 7.3% of falciparum malaria patients were admitted to ward as compared to 3.3% of *P. vivax* patients (*P *= 0.07) (Table 3). Hb levels were slightly lower in *P. falciparum* as compared to *P. vivax*, and significantly reduced in mixed-species infection. Heart and respiratory rate, blood pressure, thrombocyte count as well as creatinine and bilirubin concentrations were similar between the three types of malaria. Nonetheless, hypotension was increased in mixed-species infection and increased creatinine levels (*P *= 0.13) as well as severe thrombocytopaenia (< 50,000/µL; *P *= 0.04) were seen twice as frequently in *P. falciparum* as compared to *P. vivax* (Table 3).

Severe malaria was rare: in 32 patients (3.5%), there was evidence of severe malaria according to the WHO definition, i.e., hypotension (15; impaired perfusion not assessed), renal impairment (5), renal impairment and respiratory distress (1), severe malarial anaemia (4), prostration (3), confusion (2), jaundice (1), and abnormal bleeding (haematemesis, 1). Impaired consciousness, convulsions, hypoglycaemia, acidosis, and pulmonary oedema were not observed. Hyperparasitaemia [> 10% infected red blood cells (RBCs)] was absent, the highest parasite density observed was 148,325/µL in a patient with mixed-species infection. Parasite species did not significantly affect the proportion of severe malaria, which occurred in 3.2% (20/633), 4.1% (8/194) and 4.9% (4/82) of cases with vivax malaria, mixed-species infection and falciparum malaria, respectively (*P *= 0.64). In multivariate analysis, independent predictors of severe malaria included increasing age, female, reported diabetes mellitus, and thrombocytopaenia while increasing BMI proved to be protective (Table 4). Migration, SES, origin, previous malaria, preventive measures, and current parasite density, as well as other parameters shown in Tables 1 and 2 were not associated with severe malaria. Of the 35 (3.9%) patients who were admitted to ward, 10 were categorized as severe malaria patients. Other reasons included vomiting (5), dehydration (2), co-morbidities (2), weakness (2), suspected typhoid fever (1), jaundice (1), recent delivery (1), patient request (1), low blood pressure (1) as well as retrospectively not ascertainable causes (9). Of note, none of the patients died.

**Table 4:** Factors associated with severe malaria

Parameter	No.	% severe malaria	OR (95% CI)	P	aOR (95%)	P
Age (years)	909	na	1.04 (1.02–1.07)	0.0009	1.03 (1.00–1.06)	0.04
Gender						
Male	844	2.8	1		1	
Female	65	12.3	4.80 (1.89–11.84)	0.001	6.07 (2.32–15.89)	0.0002
Reported diabetes mellitus						
No	895	3.2	1		1	
Yes	13	23.1	8.96 (1.50–37.11)	0.009	13.60 (2.81–65.83)	0.001
BMI (kg/m^2^)	887	na	0.88 (0.76–1.00)	0.06	0.80 (0.69–0.93)	0.005
Thrombocytopaenia						
No	227	0.9	1		1	
Yes	632	4.6	5.41 (1.35–47.11)	0.01	4.64 (1.05–20.56)	0.04
Reported COPD						
No	904	3.2	1			
Yes	4	75.0	90.52 (6.86–4755.80)	0.002	–	
Construction worker/daily labourer						
No	399	4.8	1			
Yes	510	2.5	0.52 (0.24–1.13)	0.08	–	

*BMI* body mass index, *na* not applicable, *OR* odds ratio, *95% CI* 95% confidence interval, *aOR* adjusted odds ratio; n = 838, R^2^ = 0.15

## Discussion

In this study from the largest governmental hospital in urban Mangaluru, coastal southern India, almost 70% of malaria episodes were due to *P. vivax* and the vast majority of patients were managed as outpatients (96%). Severe malaria was rare (3.5%), and fatalities absent. Overall, *P. falciparum* and mixed infections showed a more pronounced manifestation than vivax malaria although respective differences were moderate. This contrasts with the severity and associated deaths of *P. vivax* infections reported from other regions of India [9–11].

The present study has several specifics and limitations, which need to be considered when interpreting the findings. Wenlock Hospital is largely attended by adult male patients of comparatively low SES, more than half of whom are construction site workers or daily labourers. This pattern in turn may impact on knowledge and awareness of malaria, degree and pace of health care utilization and thus on the clinical picture [20, 24, 25]. As a result, the manifestation of malaria presented here may differ from that encountered at private and highly modern health facilities in Mangaluru, which attract a different patient population. As Wenlock Hospital caters for the vast majority of malaria patients in the city, who are predominately socio-economically underprivileged and migrant labourers [19], the present study provides a sufficiently representative picture of malaria in Mangaluru. In the present study, the definition of severe malaria largely followed the 2014 WHO criteria [23]. Some modifications, e.g., including confusion or hypotension without evidence of reduced perfusion as indicative of severe malaria may have led to an overestimation of severe malaria. On the other hand, acidosis and hypoglycaemia could not routinely be assessed, and potentially severely sick patients attending the casualty department after outpatient hours were not regarded, which may have caused a respective underestimation. Both scenarios, however, do not explain the low rates of hospitalization and severe malaria as compared to other settings in India [6, 9–11]. Although criteria for severe vivax malaria have been formulated by WHO [23], their validity has hardly been evaluated. Although more than 900 patients were analysed, the comparatively low proportion of falciparum malaria affected the statistical power of comparisons with vivax malaria. The low proportion of children (4%) among the patients examined also needs to be considered when comparing results to those of other studies with a predominantly paediatric patient population. Lastly, co-morbidities in the present study were predominantly derived from patient statements, and 1.4% of patients reported to have diabetes mellitus. Even when considering a large proportion of undiagnosed diabetes, this contrasts with the current estimate of adult diabetes prevalence of 10.4% in India [26]. This suggests a higher proportion of co-morbidities (potentially influencing the clinical picture) than reported in the present study.

Interestingly, falciparum malaria appeared to be a local rather than an imported disease. The prevalence of *P. falciparum* mono-infection was significantly increased in construction workers and reduced in migrants from north/northeastern India, although both factors overlapped. In the latter region, the burden of malaria is increased as is the proportion of *P. falciparum* [27]. Conceivably, migrants originating from there show a higher degree of *P. falciparum*-related semi-immunity as compared to the local Mangaluru population and consequently some degree of resistance to locally transmitted *P. falciparum* parasites. Alternative explanations include an increased relapse rates in imported *P. vivax* strains, poor compliance with the 2 weeks of primaquine treatment, or an increased rate of common infectious diseases among the economically deprived migrants giving rise to an increased *P. vivax* relapse rate [28]. In these cases, migrants would show a comparatively increased proportion of *P. vivax*.

One month after the peak of precipitation, the proportion of *P. vivax* infections was lowest whereas that of *P. falciparum* was highest. In India, both temperate and tropical types of *P. vivax* relapse (i.e., long and short latency) occur [21] but for the present study, no respective information is available. Nevertheless, it is conceivable that at the beginning of peak transmission, relapses of *P. vivax* still predominated among the attending patients and *P. falciparum* proportionally increased with increasing precipitation and thus transmission.

Reported signs and symptoms did not differ substantially with parasite species even though gastrointestinal symptoms (vomiting, abdominal pain) appeared to be slightly increased in infections comprising *P. falciparum*. Nevertheless, upon clinical examination, patients with falciparum malaria showed worse general and consciousness conditions than patients with *P. vivax* or mixed infections. This may be partly due to the increased parasite counts and reduced nutritional status observed in falciparum malaria patients. On the other hand, cytokine production, potentially affecting the general condition, is higher in *P. vivax* infection than that in *P. falciparum* infections of similar parasite numbers [29, 30]. Other features of *P. falciparum* as compared to *P. vivax* infections included the highest proportion of severe thrombocytopaenia as well as trends towards more frequently increased creatinine and bilirubin levels, more anaemia, and more common hospitalization. Altogether, these differences were modest. Mixed species infection largely assumed an interim position between the manifestations of *P. vivax* and *P. falciparum*. Notable exemptions were the highest proportions of anaemia and hypotension. Given the only minor differences in the clinical picture according to parasite species, the present study does not contribute to solving the question whether mixed-species infections do rather attenuate falciparum malaria or increase its severity [4, 11, 12, 31]. One previous hospital-based study from the southern outskirts of Mangaluru conducted more than a decade ago [32] also reported a predominance of young males among the malaria patients. Based on the quantitative buffy coat (QBC) method for diagnosis, *P. falciparum* infections (34%) were more common than in the present study but mixed infections slightly less prevalent (14%). Data are not well comparable because of a considerably larger proportion of inpatients (61%) in that study but it is noteworthy that, similar to the present study, falciparum malaria was associated with increased rates of hospitalization and of altered consciousness. In the previous study from Mangaluru, two patients (1.1%) reportedly died due to cerebral malaria (no parasite species provided).

Severe malaria due to *P. vivax* infection is increasingly reported since the year 2000, and > 40% of respective studies originate from India whereas a large number of endemic countries do not report severe vivax malaria at all [6]. The inclusion of severe thrombocytopaenia as marker of severity may partially be involved in this discrepancy. In the present study, the proportion of severe vivax malaria of 3.2% would have increased to 12.9% when considering this criterion. However, abnormal bleeding (haematemesis) was observed only in one patient with *P. vivax* infection and a thrombocyte count of 78,000/µL. Other reasons for the heterogeneity of severe vivax malaria potentially include geographical differences in the peak age of vivax malaria, endemicity, chloroquine resistance, parasite virulence, and misdiagnosis of other severe diseases [6]. Beyond doubt, *P. vivax* can cause severe and fatal disease, and the existing evidence has thoroughly been reviewed [3, 4, 6, 33]. However, the reason for the respective geographical variation as well as the underlying pathophysiology are far from being understood.

Severe malaria in the present study occurred at similar proportions irrespective of parasite species. Reported diabetes mellitus was its strongest independent predictor followed by female gender, thrombocytopaenia, and increasing age, while increasing BMI was protective. Increased susceptibility to malaria in diabetic individuals has been reported from Africa [34] and there is strong evidence that diabetes increases the severity of a number of infectious diseases [35]. Female gender may correspond to peculiarities of health care utilization, and increasing age to (unreported) co-morbidities. Malnutrition, i.e., low BMI, is a known risk factor for malaria severity [36]. Thrombocytopaenia increased the risk of severe malaria almost fivefold. When replacing this factor with severe thrombocytopaenia in the multivariate model, the association weakened and remained significant only for vivax malaria (aOR, 3.5; 95% CI, 1.1–11.1). This might argue for severe thrombocytopaenia as an indicator of severe vivax malaria. However, the above estimates do not provide information on causation.

## Conclusions

Malaria in Mangaluru, coastal southern India, affects predominantly young men of low-socio-economic status, many of whom are migrant workers from other parts of India, and rarely causes hospitalization. Severe vivax malaria does occur in this setting at a rate only slightly lower than in falciparum malaria but its prevalence contrasts with substantially higher figures reported from elsewhere in the country. Studies into pathophysiology and parasite biology are needed to disentangle the respective heterogeneity.
